# Congenital Left Atrial Mitral Accessory Chord With Accessory Mitral Valve Tissue Causing Dual-Valve Regurgitation

**DOI:** 10.1016/j.jaccas.2025.106222

**Published:** 2025-12-02

**Authors:** Muhammad Salman Sabri, Fahad Waqar, Eddy Mizrahi, Andrew Fireman, Robert Watson, James L. West, Mauricio Garrido

**Affiliations:** a Department of Internal Medicine, Jefferson Abington Hospital, Abington, Pennsylvania, USA; b Department of Cardiology, Jefferson Abington Hospital, Abington, Pennsylvania, USA; c Department of Cardiac Surgery, Jefferson Abington Hospital, Abington, Pennsylvania, USA

**Keywords:** accessory mitral valve tissue, congenital cord, left atrial mitral valve accessory chord

## Abstract

**Background:**

Left atrial mitral valve (MV) accessory chord and accessory MV tissue are rare congenital anomalies that may mimic more common pathologies.

**Case Summary:**

A 30-year-old man presented with dyspnea, weight loss, and an outpatient transthoracic echocardiogram concerning for infective endocarditis. Transesophageal echocardiography revealed a chordlike structure extending from the limbus of the interatrial septum to the anterior MV leaflet, continuing through the left ventricular outflow tract, across the aortic valve, and into the ascending aorta. These findings were associated with moderate mitral regurgitation and severe aortic regurgitation. Surgical resection confirmed the diagnosis of accessory MV tissue with a left atrial MV accessory chord.

**Discussion:**

Patients may present with heart failure or malperfusion symptoms due to valvopathy or left ventricular outflow tract obstruction. Multimodal imaging and surgical resection with valve repair are key to diagnosis and management.

**Take-Home Messages:**

Chordal anomalies can mimic endocarditis and cause dual-valve disease. Early transesophageal echocardiography and surgical intervention are critical to avoid misdiagnosis.

## Visual Summary

**Figure:**
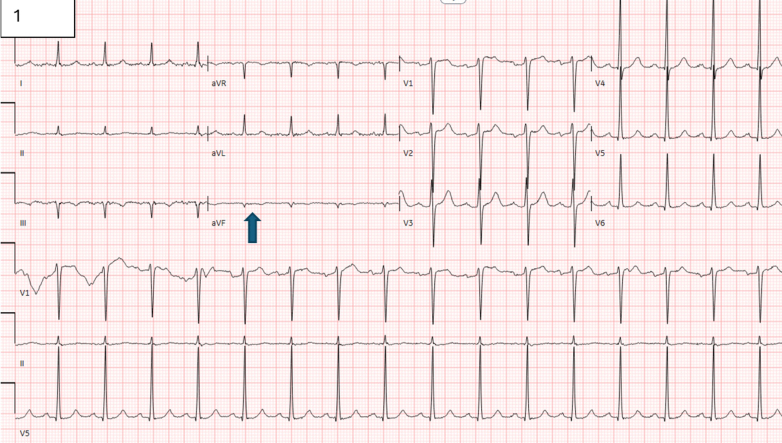

## History of presenting illness

A 30-year-old man presented after outpatient transthoracic echocardiography (TTE) concerning for mitral valve (MV) vegetation and endocarditis. Two months before arrival, he began experiencing cough, rhinorrhea, postnasal drip, and shortness of breath and was treated at an urgent care clinic with a 1-week course of amoxicillin-clavulanate and steroids for presumed pneumonia. He underwent chest radiography at that time, which was unremarkable for cardiopulmonary abnormality. Persistent symptoms led to a second urgent care visit, where he was prescribed a 2-week course of doxycycline and prednisone. Despite these treatments, he continued to experience increasing fatigue, paroxysmal nocturnal dyspnea, dry cough, intermittent facial flushing, decreased appetite, and weight loss of 10 lb in 2 months. He went to see a pulmonologist, who noted a murmur on physical examination and referred him for TTE. The study revealed a normal left ventricular (LV) ejection fraction (EF) of 60% to 65%, an LV interventricular septal diameter of 1.1 cm, a posterior wall diameter of 1.1 cm, an LV internal dimension at end-diastole of 5.7 cm, and an LV internal dimension at end-systole of 4.1 cm, with a thickened anterior MV leaflet containing a calcified structure attached to the MV annulus and extending into the LV outflow tract (LVOT), mild-to-moderate mitral regurgitation (MR) that was eccentric, and the jet that was directed posteriorly. The aortic valve (AV) was trileaflet, with a focally thickened left coronary cusp and moderate aortic regurgitation (AR) with an eccentric jet directed against the septum. The patient was advised to go to the emergency department by a pulmonologist after TTE for further evaluation because of concern for endocarditis. On presentation, vitals were stable, with a temperature of 98.8 °F, a heart rate of 84 beats/min, a blood pressure of 119/58 mm Hg, and an oxygen saturation of 99% on room air. The physical examination was remarkable for a diastolic murmur at the left sternal border; the chest was clear to auscultation; and the extremities were warm well-perfused with no splinter hemorrhages, Osler nodes, or rash.

## Past Medical History

The patient had no significant past medical or surgical history.

## Differential Diagnosis

Given the patient’s symptoms of weight loss, shortness of breath, facial flushing, cough, valvular vegetation, and insufficiency, there was concern for infective endocarditis. Other differential diagnoses included noninfective endocarditis and intracardiac tumors.

## Investigations

**Figure 1:**
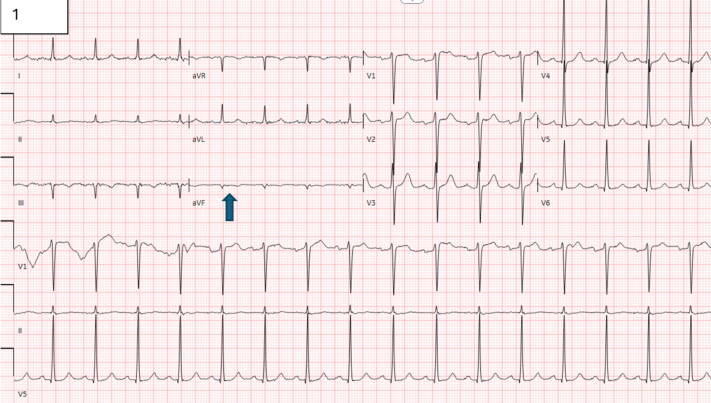
Electrocardiogram The electrocardiogram demonstrates normal sinus rhythm with Q waves in the inferior leads, indicated by the blue arrow.

The laboratory results demonstrated mildly elevated creatinine at 1.64 mg/dL and inflammatory markers (erythrocyte sedimentation rate 10 mm/h; C-reactive protein <0.3 mg/dL). The patient’s liver function, hemoglobin level, and white blood cell count were normal. Urinalysis was remarkable for trace protein and 1+ ketones, but negative for blood or leukocyte esterase. Complement (C3 and C4) levels were normal. Blood cultures were collected, which did not show any pathogen growth. Electrocardiography showed normal sinus rhythm with Q waves in the inferior leads and a QTc interval of 451 ms (Figure 1). The patient was initiated on intravenous broad-spectrum antibiotic drugs. Serologies for *Bartonella*, *Coxiella*, and *Brucella* were ordered, which did not show any growth.

**Figure 2:**
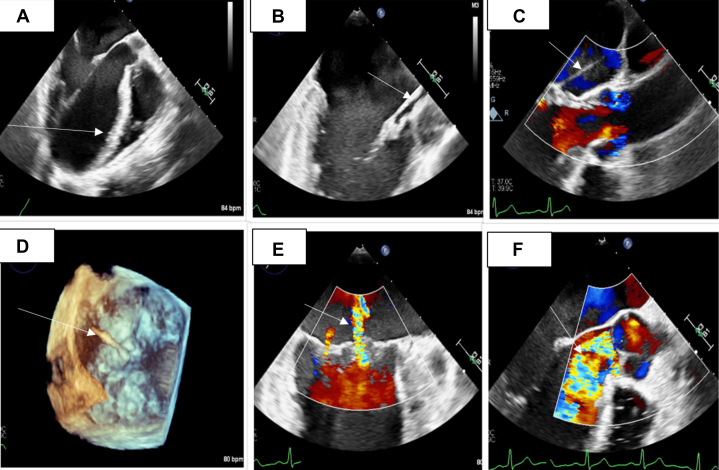
Transesophageal Echocardiogram The transesophageal echocardiogram demonstrates (A) an aberrant band of tissue (white arrow) attached to the anterior leaflet of the mitral valve at the A1/A2 region, (B, C) extending into the left atrium (white arrows). (D) The chord traverses the left ventricular outflow tract, passes through the aortic valve at the level of the left coronary cusp, and terminates in the ascending aorta (white arrow). (E) The mitral valve annulus appears dilated with mild-to-moderate mitral regurgitation (white arrow), showing both central and posterior jets. (F) Severe aortic regurgitation (white arrow) is also present, characterized by an eccentric jet and holodiastolic flow reversal in the descending thoracic aorta.

**Figure 3:**
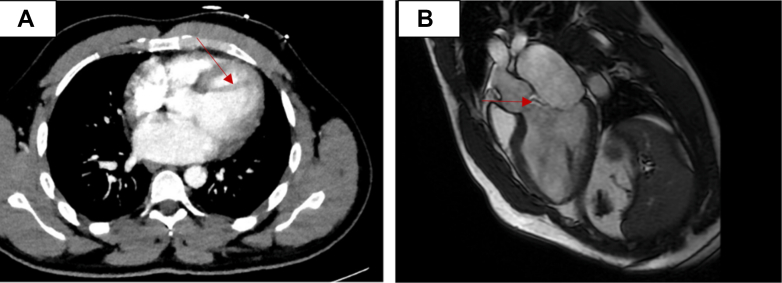
Multimodal Imaging (A) Computed tomography scan of the pulmonary arteries with intravenous contrast demonstrates a hypointense structure in the left ventricular outflow tract (red arrow). (B) Cardiac magnetic resonance scan demonstrates a mobile linear hypointense structure (red arrow) extending inferiorly from the left coronary cusp into the LVOT, which approaches the A1 scallop of the mitral valve.

Transesophageal echocardiography (TEE) (Figure 2, Video 1, Video 2, Video 3, Video 4) revealed normal LV systolic function with an EF of 60%. An aberrant band of tissue was identified, attached to the anterior leaflet of the MV at the A1 (anterior)/A2 (middle) segment, extending into the left atrium. The tissue then coursed through the LVOT, passed through the AV in contact with, but not adherent to, the left coronary cusp, and terminated in the ascending aorta. There was a dilated MV annulus along with moderate MR, with both a central and a posterior component. Severe AR was present, with an eccentric jet and holodiastolic flow reversal in the descending thoracic aorta. The right ventricle and right atrium were of normal size, with only minimal tricuspid regurgitation and no signs of pulmonary hypertension. The pulmonic valve showed normal segments with minimal pulmonic regurgitation. The aortic root at the sinuses of Valsalva was of normal size, and a very small pericardial effusion was observed, though no tamponade physiology was present. Computed tomography of the pulmonary arteries with intravenous contrast demonstrated no evidence of pulmonary thromboembolism; however, there was limited evaluation of some of the subsegmental pulmonary artery divisions due to respiratory motion artifact. It also demonstrated a hypointense structure in the LVOT (Figure 3A). Cardiac magnetic resonance demonstrated severe AR with a regurgitation fraction of 48.8% and a focal perforation of the posterior left coronary sinus along the commissure with the noncoronary sinus. It also demonstrated a mobile linear hypointense structure extending inferiorly from the left coronary cusp into the LVOT, which approaches the A1 scallop of the MV. There was no evidence of aortic paravalvular abscess. There was also evidence of moderate MR. The LV end-diastolic mass was 167.98 g; the end-diastolic volume was 348.18 mL; the end-systolic volume was 155.87 mL; the stroke volume was 192.31 mL; and the EF was 55.23%. The cardiac output was 15.96 L/min, and various body surface area–indexed values calculated included end-diastolic mass/body surface area at 79.56 g/m^2^, end-diastolic volume/body surface area at 164.91 mL/m^2^, end-systolic volume/body surface area at 73.82 mL/m^2^, stroke volume/body surface area at 91.08 mL/m^2^, and cardiac output/body surface area at 7.56 L/(min·m^2^). The LV wall thickness measurements included the anteroseptal wall thickness at 12 mm and the inferolateral wall thickness at 9 mm. Finally, the LV diameter was 73 mm at diastole and 52 mm at systole.

**Figure 4:**
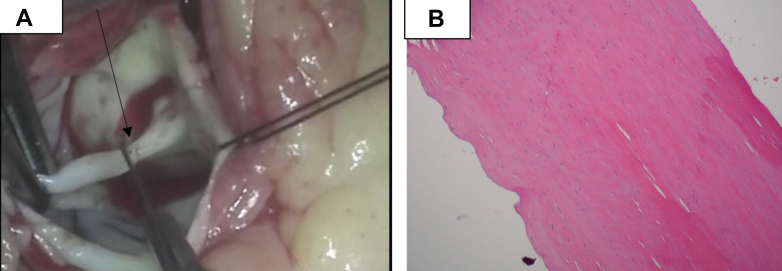
Specimen (Gross and Histopathology) (A) Intraoperative evaluation revealed a chordlike structure attached to the anterior leaflet of the mitral valve and the left coronary sinus (black arrow). (B) Histopathological analysis revealed fibrous tissue without evidence of infective endocarditis.

The patient was evaluated by the cardiothoracic surgery team and was planned for urgent AV and MV repair. The patient underwent a partial sternotomy. A midline incision was made, followed by a transverse osteotomy at the level of the fourth intercostal space. Cardiopulmonary bypass was initiated after cannulation of the aorta and common femoral vein. The del Nido cardioplegia solution was administered both antegrade and retrograde. Intraoperatively (Video 5, Video 6, Video 7), a congenital chord (Figure 4A) from the ventricular aspect of the anterior leaflet of the MV to the aortic endothelium near the left coronary os was identified and resected. A frozen section analysis showed no evidence of endocarditis. Examination of the AV revealed that the left cusp had been bisected, likely because of long-term contact with the rigid aspect of the congenital chord. The bisected left coronary cusp was reapproximated with a 5-0 Prolene suture (Ethicon). Evaluation of the MV showed no signs of endocarditis; however, a chord extending from the left atrial limbus of the fossa ovalis to the anterior MV leaflet was found and excised. Postoperative TEE demonstrated preserved LV EF, mild AR, and mild-to-moderate MR. The patient was transferred to the intensive care unit in critical but stable condition. Pathology specimens were submitted for analysis, and the estimated blood loss was 50 mL. Histopathological analysis revealed fibrous tissue with calcification (Figure 4B). Postoperatively, the patient was initiated on coumarin, metoprolol succinate, and losartan. He was subsequently discharged to a rehabilitation facility, with plans for outpatient follow-up.

## Discussion

The left atrial MV accessory chord is a rare congenital anomaly, with an incidence of 2% in autopsy reports,^1^ with around 4 cases in 6,500 autopsies.^2^ This can be found concomitantly with accessory MV tissue (AMVT), which is also a rare congenital anomaly, with an incidence of 1:26,000 echocardiograms in the adult population.^3^ Our patient likely had AMVT with a chordal attachment between the anterior MV leaflet and the left coronary cusp, with an additional left atrial MV accessory chord extending from the mitral leaflet to the limbus of the interatrial septum.

It is hypothesized that the left atrial MV accessory chord results from a developmental defect in the endocardial cushion in the second to third week of the first trimester.^1^ These bands are believed to be remnants of the septum primum, a derivative of the endocardial cushion, showing a leftward deviation and extension.^1^ Atrioventricular valves, such as MV, also derived from the endocardial cushion, and AMVT seems to be related to the abnormal and incomplete separation of the MV from the endocardial cushion during cardiac development.^3^ Our patient likely had a congenital endocardial cushion defect, which led to AMVT and the left atrial accessory MV chord.

The left atrial MV accessory chord may present concurrently with other cardiac conditions, such as patent foramen ovale, Chiari network, or cor triatriatum dexter.^1^ Patients with left atrial MV accessory chordae have been diagnosed between the ages of 15 and 85 years;^1^ however, when associated with AMVT, diagnosis often occurs in the second or third decade of life because of the onset of symptoms of valvopathy.^2^^,^^3^ A patient found to have a left atrial MV accessory chord incidentally usually has a benign finding unless there is attachment of the chord to the MV, which can cause mild-to-severe MR and associated complications, such as infective endocarditis.^1^^,^^2^ When associated with AMVT, there can be LVOT obstruction, malperfusion (chest pain, presyncope, or syncope), or volume overload symptoms (shortness of breath, leg swelling, orthopnea, bendopnea, anorexia, and weight loss, as seen in our patient with chronic gut edema) from chord attachment extending from the MV leaflet to the LVOT and aorta, such as left coronary cusp in our case.^3^ AMVT with a chord extending into the LVOT and attaching to the AV leaflet, coronary sinus, or aortic wall can result in either bowing of the AV leaflet toward the LV or long-term trauma to the AV leaflet through bypass of the chord-like structure through the AV, as seen in our case—both of which can lead to AR. There can also be localized complications, such as endocarditis, or systemic complications, including cardioembolic events due to increased mobility of the chordal structure.^3^ The accessory chord most commonly attaches to the anterior MV leaflet within the left atrium, though attachment to the posterior leaflet has also been reported.^1^ Anomalous chordae that attach to an AV valve cusp can cause AR.^4^ In addition, there are reported associations between anomalous chordae and the bicuspid AV.^5^

When evaluating chordlike structures extending from the MV to the LVOT and left atrium, the differential diagnosis includes left atrial MV accessory chord, AMVT, thrombus, vegetation, tumor, chordae tendineae, and cor triatriatum.^3^ Additional considerations include LV free tendons, though these typically extend from the interventricular septum to either a papillary muscle or the LV free wall.^6^ Echocardiography is the diagnostic modality of choice for identifying AMVT and left atrial MV accessory chordae.^3^ TEE offers advantages over TTE by providing superior visualization of the chordal structure’s attachment, extension, associated complications, and morphology.^3^ In addition, TEE can help differentiate chordal abnormalities from cardiac tumors, such as myxomas,^7^^,^^8^ thrombus, and vegetations, and more accurately assess for obstruction, flow acceleration, or other hemodynamic changes.^3^ Further imaging, including computed tomography and cardiac magnetic resonance, can be considered to delineate the chordal anatomy and aid in preoperative planning, as in our case.

Asymptomatic patients with a left atrial MV accessory chord or AMVT without significant valvular disease generally do not require further evaluation.^1^ In cases of mild-to-moderate MR, serial echocardiographic monitoring is recommended to track disease progression and guide clinical management.^1^ Surgical intervention is indicated for patients with significant MR, AR, or infective endocarditis.^1^^,^^3^ MV and AV repair with resection of the accessory chord is typically the preferred treatment approach.^3^

## Conclusions

This case depicts a rare presentation of a left atrial MV accessory chord with associated AMVT, which led to AV leaflet trauma, resulting in severe AR in a young patient who presented with reports of heart failure and concern for endocarditis. Surgical resection with valve repair helped in tissue diagnosis, and improvement in AR, with symptomatic benefit.

**Take-Home Messages**

• Left atrial mitral valve accessory chordae and accessory mitral valve tissue are among the differential diagnoses in patients with symptoms of heart failure and concern for endocarditis on transthoracic echocardiography.
• Multimodality imaging, including transesophageal echocardiography and cardiac magnetic resonance, along with surgical resection is essential for diagnosis.
• Early recognition and surgical management are key to preventing long-term complications such as severe regurgitation, cardiomyopathy, endocarditis, and embolic events.

## Funding Support and Author Disclosures

The authors have reported that they have no relationships relevant to the contents of this paper to disclose.

## Appendix

Video 1 Transesophageal Echocardiogram Shows an Aberrant Band of Tissue Arising From the Anterior Mitral Leaflet at the A1/A2 Region, Extending Into the Left Atrium It then courses through the left ventricular outflow tract and crosses the aortic valve, coming into contact with, but not adherent to, the left coronary cusp.

Video 2 Transesophageal Echocardiogram Reveals a Chord Projecting Into the Left Ventricular Outflow Tract, Traversing the Aortic Valve at the Level of the Left Coronary Cusp, and Extending Into the Ascending Aorta

Video 3 Transesophageal Echocardiogram Demonstrates Mild-to-Moderate Mitral Regurgitation With Central and Posterior Jets

Video 4 Transesophageal Echocardiogram Demonstrates Aortic Regurgitation With an Eccentric Jet Across the Left Ventricle Through the Aortic Valve

Video 5 Intraoperative Surgical Specimen Reveals a Chord Attached to the Anterior Leaflet of the Mitral Valve, Extending Through the Left Ventricular Outflow Tract and Aortic Valve, and Inserting Into the Aortic Wall at the Level of the Left Coronary os The chord appears calcified below the aortic valve and pliable above it.

Video 6 Intraoperative Video Shows a Tear in the Midportion of the Left Coronary Cusp, Resulting From Chronic Trauma Caused by the Calcified Chord

Video 7 Aortic Valve Was Approximated and Successfully Repaired Using a 5-0 Prolene Suture, Resulting in an Improvement in Aortic Regurgitation From Severe to Mild

## References

[1] Sahebjam M., Yadegar A., Farmanesh M. (2024). Left atrial mitral valve cord: unveiling complexity through advanced 3D TEE imaging - a case report. Radiol Case Rep.

[2] Patrianakos A.P., Zacharaki A.A., Marketou M.E., Parthenakis F.I. (2020). Anomalous left atrial chord as a rare cause of mitral regurgitation. Eur Heart J Case Rep.

[3] Gurrieri C., Nelson J., Wurm H., Cicek M.S., Maalouf J.F. (2019). An extremely rare cause of mitral regurgitation-accessory commissural mitral tissue with anomalous left atrial chordal attachment. CASE (Phila).

[4] Mochizuki Y., Yamasaki M., Fujiwara R., Nakagiri K., Iwahashi K., Shite J. (2018). Severe aortic regurgitation caused by congenital quadricuspid aortic valve with an anomalous cord. Can J Cardiol.

[5] Vowels T., Gonzalez-Stawinski G., Ko J. (2014). Anomalous cord from the raphe of a congenitally bicuspid aortic valve to the aortic wall producing either acute or chronic aortic regurgitation. J Am Coll Cardiol.

[6] Liu Q., Wang Y., Ehdaie A. (2023). False tendons in the left ventricle: implications for successful ablation of left posterior fascicular tachycardias. J Am Coll Cardiol.

[7] Sabri M., Talukder Z., Ijaz N. (2025). Uncharted territory: the first case of cardiac myxoma in a patient with NF2. JACC Case Rep.

[8] Sabri M., Qamar S., Ijaz N. (2024). Embolic stroke in a patient with left atrial myxoma. JACC Case Rep.

